# Nematocidal activity of fungal filtrates on eggs and juveniles of five species of sedentary endoparasitic nematodes

**DOI:** 10.2478/jofnem-2025-0018

**Published:** 2025-06-04

**Authors:** B. Jumbam, A. B. Peetz, V. S. Kunwar, L. Zhang, I. A. Zasada, M. C. Aime

**Affiliations:** Department of Plant Sciences and Plant Pathology, Montana State University, Bozeman, MT 59715.; Department of Botany and Plant Pathology, Purdue University, West Lafayette, IN 47907.; USDA-ARS Horticultural Crops Disease and Pest Management Research Unit, Corvallis, OR 97330.

**Keywords:** Biological control, cyst nematodes, fungal filtrates, root knot nematodes, secondary metabolites

## Abstract

Research efforts are needed to develop new biocontrol strategies against plant-parasitic nematodes (PPNs) to replace chemicals and maintain sustainable crop production. In this study, filtrates obtained from fungi isolated from cyst nematodes (Heteroderidae) were evaluated for activity against eggs and second-stage juveniles (J2) of five PPNs: *Globdera ellingtonae, Heterodera glycines, Meloidogyne incognita*, *M. hapla*, and *M. chitwoodi*. Initially, filtrates of 42 fungal isolates were evaluated for effects on *G. ellingtonae* and *H. glycines* egg viability. After the initial screening, six of the fungal isolates were selected for further evaluation against additional PPN eggs and J2 based upon evidence of usage in other studies, fast growth, and frequency of isolation. Filtrates from *Alternaria tenuissima* JB217, *Fusarium acaciae-mearnsii* JB201, *Purpureocillium lilacinum* JB209, and *Trichoderma virens* JB98 reduced *H. glycines* egg viability by >80%. *Aureobasium* sp. JB70, *F. proliferatum* JB173, and *P. lilacinum* JB209 reduced *G. ellingtonae* egg viability by >50% but had negligible effect on the J2 stage of this nematode. Filtrate from *F. acaciae-mearnsii* JB201was the most lethal against PPNs, immobilizing ~100% of J2 of all nematode species while filtrate from *A. tenuissima* JB217 only immobilized J2 of *G. ellingtonae, M. chitwoodi*, and *M. hapla*. These fungal filtrates are therefore promising alternative sources of natural bioactive substances for the potential management of PPNs.

Plant-parasitic nematodes (PPNs) are among the most damaging plant pathogens affecting the yield of leguminous crops such as soybean, tubers such as potato, and cash crops such as cotton, globally (Brown, 1969; Greco and Moreno, 1992; Robinson, 2007; Wrather and Koenning, 2009; Zasada et al., 2019). Prior to the emergence of the unpleasant side effects of chemical agents in the 1970s, PPNs were managed primarily with chemical nematicides (Desaeger et al., 2020; Thrupp, 1991). With knowledge about their toxicity and the consequent health and environmental concerns, some of the most effective chemical nematicides (including methyl bromide, fenamiphos, and aldicarb) were restricted by several countries (Cone, 2010; Desaeger et al., 2020; EPA, 2010; Ristaino and Thomas, 1997; Xiang et al., 2017a). This led to a decrease in the number of chemical nematicides available for nematode management in North America and the world.

The development of microbial biocontrol products for the management of PPNs is an alternative to the use of chemical nematicides (Desaeger et al., 2020). Fungi and bacteria are among the most widely used microbes in biocontrol applications against PPNs (Chen and Liu, 2005; Di Marco et al., 2022; Liu and Chen, 2000; Nitao et al., 2002; Yao et al., 2023). The utilization of fungal biocontrol agents—formulated as spores, for instance—has drawn the interest of many growers as a substitute for chemical applications (Berg, 2009). Dackman (1990) outlined the limitations of direct application of microbes in agricultural fields. Consequently, agricultural industries are formulating biopesticides from microbes and their metabolites in partnership with research institutions (Migunova and Sasanelli, 2021). There is a continuing need to identify and develop additional biocontrol agents for PPN management.

To expand the effort of identifying potential biocontrol agents, in a previous study, we cultured and identified ~300 isolates of fungi from 49 cysts nematode (belonging to *Heterodera* and *Globodera* spp.) populations collected from around the world (Jumbam et al., 2024). These isolates represented 59 fungal species, of which 35 were first-time associates of cyst nematodes. The new fungal associates included *Alternaria tenuissima, Aspergillus ceber, Aureobasidium* sp., *Debaryomyces hansenii*, several species of *Fusarium* (*F. acacia-mearnsii, F. proliferatum, F. pseudograminearum*, and *F. tricinctum*), *Pseudogymnoascus* sp., and *T. virens*. Apart from *A. tenuissima* and *Pseudogymnoascus* sp., whose ability to suppress PPNs is currently unknown, species of *Aureobasidium* (Di francesco et al., 2020), *Aspergillus* (Kagot et al., 2019), and *Fusarium* (Sajeena et al., 2020) have been reported as biocontrol agents of nematodes. Therefore, in this study isolates belonging to these genera and others that have not been previously reported in biocontrol studies were selected and screened for nematocidal activity against eggs and second-stage juveniles (J2) of five endoparasitic sedentary nematodes.

The objectives of this research were to (i) assess filtrates of diverse culturable fungi for nematocidal activity on eggs of *G. ellingtonae* and *H. glycines*, and (ii) evaluate the nematocidal efficacy of filtrates on J2 of *G. ellingtonae*, *M. chitwoodi, M. hapla*, and *M. incognita*. Fungal filtrates were utilized to conduct these in vitro investigations to address the hypothesis that the tested fungal filtrates contain nematocidal secondary metabolites.

## Materials and Methods

### Preparation of plant-parasitic nematode inoculum

Healthy cysts of *G. ellingtonae* (originally collected from Powell Butte, OR) and *H. glycines* (originally collected from West Lafayette, IN) were handpicked under a dissecting microscope. Egg suspensions were prepared by selecting 100 healthy cysts that were then submerged in a 2% sodium hypochlorite (NaOCl) solution for 1–2 min to surface-disinfect and removal of contaminants. Cysts were then rinsed 4–5 times with sterile distilled water (dH2O) before crushing with a melted pipette tip in a 2 ml tube. The mixture was transferred onto a 25 μm sieve, rinsed twice with 2 ml of dH2O, then washed into a 50 ml tube. Removal of cyst debris was done by sugar centrifugation (Jenkins, 1964). The resulting egg suspension was poured onto a 25 μm sieve and rinsed with dH2O. Finally, the eggs were transferred into a 15 ml tube and the volume was adjusted to 7 ml with dH2O and used in assays.

Second-stage juveniles of various PPNs were also collected. To obtain *G. elllingtonae* J2, 10–20 healthy cysts were transferred to a 100 ml beaker containing 2–3 ml of potato root diffusate (PRD):water at a concentration of 1:5 or 1:10 and then wrapped with aluminum foil and incubated at 25°C (Zasada et al., 2013). Hatched *G. ellingtonae* J2 were collected daily starting at day three and stored at 4°C until use. *Meloidogyne chitwoodi, M. hapla*, and *M. incognita* were also considered in the study. For each species, cultures were established by planting a 3-weeks-old tomato *(Solanum lycopersicum)* “Rutgers” seedling in infested soil collected from a potato field in Washington *(M. chitwoodi*), a vineyard in Washington *(M. hapla)*, and a vineyard in California *(M. incognita).* Plants were grown for a minimum of 9 wk under greenhouse conditions with 16 hr daylight, an average of 22°C, and fertilized weekly with 20-20-20 (J.R. Peters, Allentown, PA). Single egg masses were handpicked from each destructively harvested culture plant and used to inoculate new seedlings grown in a pasteurized 1:1 sand:loam mix and grown again under greenhouse conditions for a minimum of nine weeks. Species identification for each population was confirmed through molecular analysis of the ribosomal internal transcribed spacer (ITS) region by the North Carolina Department of Agriculture and Consumer Services (Raleigh, NC). To obtain *Meloidogyne* spp. J2, egg masses were handpicked into a 5% NaOCl solution, shaken for 3 min at 300 rpm, and collected on a 25 μm sieve. The resulting surface-sterilized eggs were placed on a 1.5 cm hatching chamber with 30 μm nylon mesh (modified from Zasada et al., 2006). Hatching was achieved as indicated above but autoclaved tap water was used as the hatching medium for *Meloidogyne* spp. Hatched *Meloidogyne* J2 were collected daily and stored at 4°C until use.

### Fungal isolates and filtrate preparation

The fungi used in this study were originally isolated and characterized as described by Jumbam et al. (2024). Briefly, *Globodera* spp. cysts were collected from the field or from greenhouse cultures in France, Peru, U.K., and the U.S. *Heterodera* spp. cysts were collected from the field and from greenhouse cultures in the U.S. Cysts were sent to Purdue University, Lafayette, IN where they were used to culture fungi on semi-selective media including potato dextrose agar (PDA: BD Difco^TM^, Fisher Scientific, Leicestershire, U.K.), yeast malt extract agar (YMA: ThermoFisher Scientific, Waltham, MA), and rose bengal agar (RBA: Fisher Scientific, Berkeley, MO). Plates, 60 × 15 mm, were amended with chloramphenicol at 250 mg/L, incubated at 28°C, and sub-cultured until pure cultures were obtained and identified by ITS sequencing. Isolates were preserved on PDA slants at 4°C and glycerol at −80°C. From this library of fungi, selections for inclusion in the study were identified using three criteria. The first criterion was evidence of fungal usage in previous studies (Kerry, 1980; Chen et al., 1996). Secondly, fungi that were fast-growing were selected. Finally, those that were isolated most frequently as well as those that were isolated for the first time and previously shown to have biocontrol activity in similar research studies were included.

Fungal filtrates were prepared from 10-days-old fungal cultures following Meyer et al. (2004) with slight modifications. Briefly, a small quantity of the mycelium was scraped with a sterile toothpick and placed into a 15 ml tube. The volume was adjusted to 12 ml using Czapek broth (35 g/mL: BD Difco, ThermoFisher Scientific, Waltham, MA) (Jumbam, 2022) and the mixture was vigorously shaken and incubated at 25°C, agitating every two days. After 14 days of incubation, the mixture was passed through a 25 μm sieve to remove mycelial contaminants. The suspension was then centrifuged at 14,000 rpm for 5 min in an Eppendorf 5430 centrifuge (Eppendorf AG, Hamburg, Germany) and the supernatant (filtrate) was collected. The filtrate was further inspected under a light microscope (400x) for the presence of fungal structures (contaminants). Any supplementary fungal structures were removed by passing the solution through a 0.45 μm syringe filter (ThermoFisher Scientific). The purified fungal filtrates were then stored at 4°C until use.

### Initial screening of fungal filtrates against *H. glycines* and *G. ellingtonae* eggs

Forty-two fungal isolates were screened for biocontrol activity on *H. glycines* and *G. ellingtonae* eggs (Table 1). An aliquot of 50 μl of *H. glycines* or *G. ellingtonae* egg suspension containing approximately 100 eggs was pipetted into a 1.5 ml tube containing 100 μl of the fungal filtrates; dH2O was included as a control. Each fungal filtrate treatment was replicated three times for each nematode species. The tubes were incubated for five days. At this time, 25 μg/mL of Acridine orange (AO; a fluorescent dye that differentiates live from dead eggs) was added (Pillai and Dandurand, 2019) and tubes were incubated at room temperature for 4 hr. The tubes were then centrifuged for 90 sec at 3000 rpm and the supernatant was discarded. One hundred μl of dH20 was added to the tubes and they were centrifuged again for 90 sec to remove excess dye from eggs. The supernatant was discarded and 50 μl of dH2O was then added to resuspend the nematode eggs. *Globodera ellingtonae* egg viability was evaluated using an Echo Revolve fluorescent microscope (ECHO Laboratories, San Diego, CA) with the 4′,6-diamidino-2-phenylindole (DAPI) parameter to determine egg viability. For *H. glycines*, an Olympus BX43F with a DP80 camera attachment (Olympus Bartlett, TN) was used with Bright Field (BF), Tetramethylrhodamine-5-(and 6)-isothiocyanate (TRITC), and Fluorescein isothiocyanate (FITC) parameters to determine egg viability. Unstained eggs were considered as intact (live). Filtrate toxicity was scored as: Percentage egg viability = [1 – (stained eggs/total number of eggs observed)] *100. From this initial screening, six fungi were retained for additional assays against other PPNs and life stages (Table 2).

**Table 1:** Fungi isolated from cyst nematodes initially screened for impacts on egg viability of *Globodera ellingtonae* (*Ge*) and *Heterodera glycines* (*Hg*). More information on isolation of these strains can be found in Jumbam et al. (2024).

**Fungal Species**	**Strain**	**Source^a^**	**Site^b^**	**Country**	**% viable^c^**	
					**Hg**	**Ge**
*Alternaria* sp.	JB176	*Gp*	GH	USA	-	53
*A. tenuissima*	JB217	*Hg*	FD	USA	0	-
*Arthopyrenia salicis*	JB155	*Gr*	GH	France	67	0
*Aureobasidium* sp.	MCA7097	*Air*	FD	Peru	-	17
*Aureobasidium* sp.	JB70	*Ge*	FD	USA	-	29
*Cephalotrichum microsporum*	JB178	*Gr*	GH	France	-	20
*Cladorrhinum samala*	JB211	*Hg*	FD	USA	67	-
*C. cladosporioides*	JB93	*Ge*	FD	USA	-	46
*C. herbarum*	JB179	*Gp*	GH	France	-	18
*Cladosporium* sp.	JB110	*Gp*	GH	UK	-	42
*Colletotrichum nigrum*	JB115	*Gr*	GH	USA	100	-
*Fusarium acacia-mearnsii*	JB201	*Hg*	FD	USA	0	-
*F. culmorum*	JB121	*Ha*	GH	USA	69	-
*F. equiseti*	JB214	*Hg*	FD	USA	80	-
*F. equiseti*	JB126	*Gr*	FD	USA	-	36
*F. oxysporium*	JB213	*Hg*	FD	USA	81	50
*F. proliferatum*	JB173	*Gp*	GH	USA	82	1
*F. pseudograminearum*	JB276	*Gs*	FD	Peru	-	2
*F. redolens*	JB280	*Gs*	FD	Peru	-	8
*Fusarium* sp.	JB266	*Gs*	FD	Peru		48
*F. tricinctum*	JB124	*Ha*	GH	USA	57	-
*Geomyces* sp.1	JB219	*Ha*	FD	USA	100	-
*Geomyces* sp.2	JB220	*Ha*	FD	USA	67	-
*Humicola grisea*	JB275	*Gs*	FD	Peru	-	48
*Keithomyces carneus*	JB282	*Gs*	FD	Peru	-	37
*Neocosmospora solani*	JB123	*Gr*	GH	France	67	46
*N. solani*	JB202	*Hg*	FD	USA	60	-
*Penicillium janthinellum*	JB122	*Gr*	FD	USA	-	10
*P. rubens*	JB270	*Gs*	FD	Peru	-	47
*Penicillium* sp.	JB204	*Hg*	FD	USA	0	-
*Purpureocillium lilacinum*	JB209	*Hg*	FD	USA	83	-
*Thielavia* sp.	JB104	*Ha*	GH	USA	100	-
*Trichocladium griseum*	JB274	*Gs*	FD	Peru	-	47
*Trichoderma* sp.1	JB96	*Gr*	GH	USA	41	-
*Trichoderma* sp.2	JB98	*Ha*	GH	USA	11	28
*T. virens*	JB101	*Gr*	GH	France	71	45
*Unknown1*	JB240	*Ha*	FD	USA	70	-
*Unknown2*	JB243	*Ha*	FD	USA	87	-
*Unknown3*	JB245	*Ha*	FD	USA	0	-
*Unseq2*	JB278	*Gr*	FD	USA	-	42
*Unseq3*	JB285	*Gs*	FD	Peru	-	35
*Zopfiella longicaudata*	JB100	*Gp*	FD	France	-	45
*Control*	*-*	*-*	-	-	73	51

a *Ge = G. ellingtonae*, *Gp = Globodera pallida*, *Gr = Globodera rostochiensis*, *Gs = Globodera* sp., *Ha = Heterodera avenae*, *Hg = H. glycines*.

b FD = field, GH = greenhouse.

c Egg viability was determined by staining eggs with Acridine orange followed by microscopic evaluation.

**Table 2:** Top six fungi retained for fungal filtrate assays against *Heterodera glycines (Hg), Globerdera ellingtonae (Ge), Meloidogyne chitwoodi (Mc), M. hapla* (*Mh*), and *M. incognita* (*Mi*) eggs or second-stage juveniles (J2).

**Strain**	**Fungal Species**	**Nematode**	**Life stage assayed**
JB70	*Aureobasidium sp.*	*Ge*	eggs
JB98	*Trichoderma virens*	*Ge, Hg*	eggs
		*Ge, Mc, Mh, Mi*	J2
JB173	*Fusarium proliferatum*	*Ge*	eggs
JB201	*F. acaciae-mearnsii*	*Hg*	eggs
		*Ge, Mc, Mh, Mi*	J2
JB209	*Purpureocilium lilacinum*	*Ge, Hg*	eggs
		*Ge, Mc, Mh, Mi*	J2
JB217	*Alternaria tenuissima*	*Hg*	eggs
		*Ge, Mc, Mh, Mi*	J2

### Activity of retained fungal filtrates against *G. ellingtonae* and *H. glycines* eggs

The same experimental setup as in the initial screening above was used to test the effect of the retained fungal filtrates on *G. ellingtonae* egg viability. To evaluate the impact of fungal filtrates on *H. glycines*, an egg viability assay was performed in a 24-well flat bottom plate (VWR, PA, USA) according to Meyer et al. (2004). Each well received a total of 400 μl of fungal filtrate and egg suspension (~100 eggs) combined in a 1:1 (v/v) ratio; the combination of dH2O and egg suspension served as the control. Plates were sealed with parafilm and incubated in the dark at room temperature and monitored using a Stemi 508 stereomicroscope (Zeiss, Gottingen, Germany). Treatments were replicated five times for both nematode species, and the experiments were conducted twice. Egg viability was evaluated at 3, 5, and 7 days post-inoculation. *G. ellingtonae* egg viability was assessed using the staining approach described above and *H. glycines* egg viability was evaluated by identifying structural aberrations (“vacuole-like” structures). The presence of vacuolar aberrations indicated nonviability and was estimated as: Percentage viability = [1 – (number of stained/aberrated eggs in a well/total number of eggs observed)] *100.

### Activity of retained fungal filtrates against *G. ellingtonae* and *Meloidogyne* spp. J2

Assays to determine the activity of the top six fungal filtrates (Table 2) against PPN J2 were conducted following Meyer et al. (2004), with modifications. For *G. ellingtonae*, hatched J2 were washed with dH2O over a 25 μm sieve and collected into a 15 mL tube. The volume was adjusted to one *G. ellingtonae* J2/μl. To each well of a 96-well flat bottom plate (VWR) 100 ul of fungal filtrate, Czapek broth (control), or dH2O (control) was added followed by 100 μl of *G. ellingtonae* J2 suspension. Plates were then sealed, covered in foil, and incubated at 25°C in the dark for 24 hr. Treatments were replicated three times and the experiment was repeated. Motility of *G. ellingtonae* J2 was determined following a modified technique from Chen and Dickson (2000). Briefly, 10 μl of 1 M NaOH was added to each well. After 5 min of exposure to the alkaline solution, mobile *G. ellingtonae* J2 changed their shape while immobile *G. ellingtonae* J2 were static, lying straight in the filtrate medium. The plates were observed under an inverted Olympus CKX53 microscope at 100 x magnification (Olympus, Center Valley, PA, USA).

For *Meloidogyne* J2 assays, approximately 100 J2 in 100 μl dH20 were added to 100 μl of each fungal filtrate in a 96-well flat bottom plate. The lid was sealed using parafilm and the plate was incubated for 24 hr in the dark at 25°C. After this period, the NaOH method was used to determine the motility of *Meloidogyne* J2. Treatments were replicated four times, and the experiment was conducted twice. For all of the PPNs, percentage mobile J2 = [1 – (immobile J2/total number of J2 observed)] *100.

### Statistical analysis

All data were subjected to analysis of variance (ANOVA) using the R software v.4.3.2 (R Core Team, 2021). Data were inspected for normality and homogeneity of variance using Shapiro-Wilk’s and Levene’s tests, respectively (Levene, 1960; Shapiro and Wilk, 1965). A significant result for Shapiro’s test indicates a violation of normality while a non-significant Levene’s test indicates that variances are equal, and the assumption is met. Non-normally distributed data were analyzed using the Kruskal-Wallis rank sum test (Kruskal and Wallis, 1952). Treatment means were either compared by Tukey HSD (honestly significant difference) or Wilcoxon’s test (Wilcoxon, 1945; Keselman and Rogan, 1977) for normal and non-normal data respectively (alpha = 0.05) and p-values were adjusted according to Benjamini and Hochberg (Benjamini and Hochberg, 1995).

## Results

### Initial screening of fungal filtrates for activity against *H. glycines* and *G. ellingtonae* eggs

**Figure 1:**
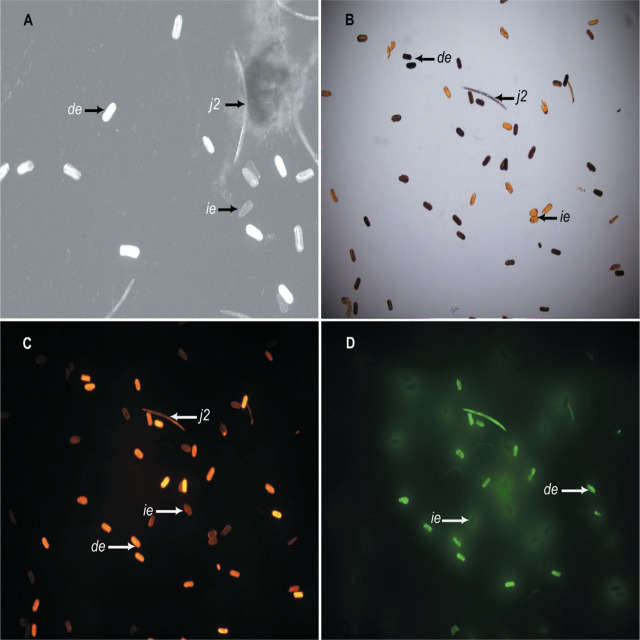
Micrograph of parameters used to differentiate between intact and damaged *Heterodera gycines* and *Globodera ellingtonae* eggs. A) 4′,6-diamidino-2-phenylindole (DAPI) stained eggs viewed under the Echo Revolve fluorescent microscope; B) Bright field view with the Olympus microscope; C) Tetramethylrhodamine-5-(and 6)-isothiocyanate (TRITC) view with the Olympus microscope; and D) Fluorescein isothiocyanate (FITC) view with the Olympus microscope (*ie* = intact eggs, *de* = damaged eggs, and *j2* = second-stage juveniles).

Filtrates that reduced *G. ellingtonae* egg viability by >70% were from *Arthopyrenia salicis* JB155, *Aureobasidium* sp. JB70, *Cephalotrichum microsporum* JB178, *Cladosporium herbarum* JB179, *F. profliferatum* JB173, *F. pseudograminearum* JB276, *F. redolens* JB280, *Penicillium janthinellum* JB122, and *T. virens* JB98 (Table 1). Damaged *G. ellingtonae* eggs retained the AO stain, while intact (live) eggs were negative for the dye. Stained eggs appeared white under the Revolve microscope while the unstained eggs were grayish to almost transparent (Fig. 1A).

Among the fungal filtrates tested against *H. glycines* eggs, *A. tenuissima* JB217, *F. acacia-mearnsii* JB201, *Penicillium* sp. JB204, *T. virens* JB98, and an unidentified fungal strain JB285 (Unknown3) reduced egg viability by >70% (Table 1). Under the Olympus BX43F, *H. glycines* eggs were observed using three sensors including BF, TRITC, and FITC (Figs. 1B–D, respectively). The contrast between these three sensors helped to capture most of the stained (dead) eggs. Based upon the initial screening, six fungal isolates including *A. tenuissima* JB217, *Aureobasidium* sp. JB70, *F. acaciae-mearnsii* JB201, *F. proliferatum* JB173, *P. lilacinum* JB209, and *T. virens* JB98 were selected for further assays against the five sedentary endoparasitic nematodes (Table 2).

### Activity of retained fungal filtrates against *G. ellingtonae* and *H. glycines* eggs

**Figure 2:**
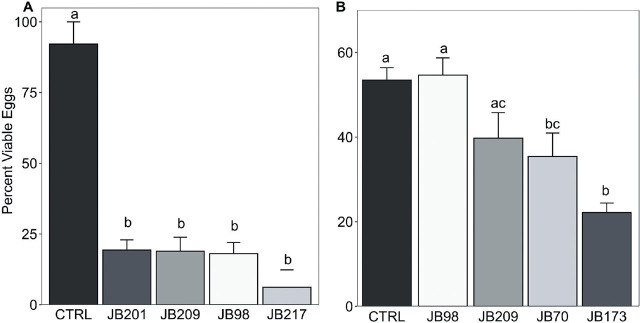
Effect of fungal filtrates on A) *Heterodera glycines* and B) *Globodera ellingtonae* eggs. Bars within a nematode species followed by the same letter are not significantly different from each other (*P* ≥ 0.05). Control (CTRL), *Fusarium acaciae-mearnsii* (JB201), *Purpureocillium lilacinum* (JB209), *Trichoderma virens* (JB98), *Alternaria tenuissima* (JB217), *Aureobasidium* sp. (JB70), and *Fusarium proliferatum* (JB173).

There was no impact of time (3, 5, or 7 days) on egg viability, therefore, data was combined for analysis. *Alternaria tenuissima* JB217, *F. acaciae-mearnsii* JB201, *P. lilacinum* JB209, and *T. virens* JB98 filtrates were evaluated against *H. glycines* eggs (Fig. 2A). All these fungal filtrates reduced egg viability by > 80%. While there was no difference in egg viability among the fungal filtrates, viability was significantly reduced with the fungal filtrates compared to the control (χ^2^(4) = 18.17, p = 0.001). Filtrates of *Aureobasidium* sp. JB70, *T. virens* JB98, *F. proliferatum* JB173, and *P. lilacinum* JB209 were evaluated against *G. ellingtonae* eggs (Fig. 2B). *Aureobasidium* sp. JB70 and *F. proliferatum* JB173 reduced *G. ellingtonae* egg viability by 29 and 41%, respectively, compared to *T. virens* JB98 and the control (χ^2^(4) = 25.03, p < .001). *Fusarium proliferatum* JB173 had a significantly higher nematocidal effect on *G. ellingtonae* eggs than *P. lilacinum* JB209. There was no significant difference in *G. ellingtonae* egg viability effects between filtrates from *T. virens* JB98, the control, and *P. lilacinum* JB209 (Fig. 2B).

### Activity of retained fungal filtrates against *G. ellingtonae* and *Meloidogyne* spp. J2

**Figure 3:**
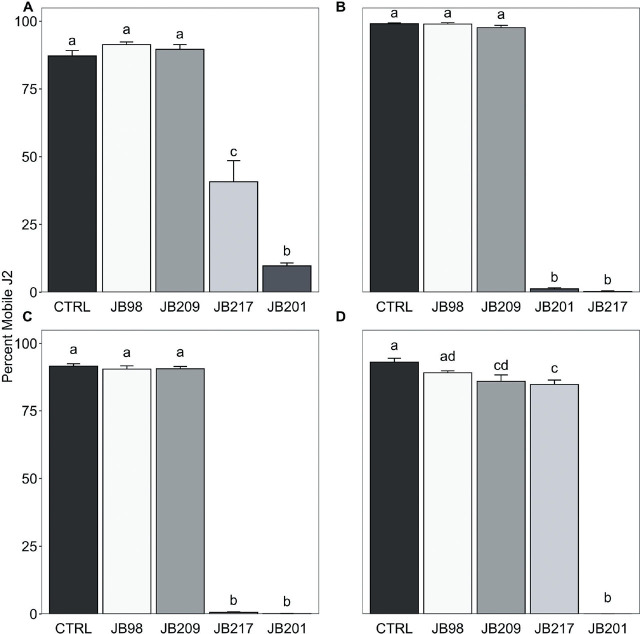
Effect of fungal filtrates on A) *Globodera ellingtonae* (*Ge*), B) *Meloidogyne chitwoodi* (*Mc*), C) *M. hapla* (*Mh*), and D) *M. incognita* (*Mi*) second-stage juvenile (J2) mortality. Bars within a nematode species followed by the same letter are not significantly different from each other (*P* ≥ 0.05). Control (CTRL), *Fusarium acaciae-mearnsii* (JB201), *Purpureocillium lilacinum* (JB209), *Trichoderma virens* (JB98), and *Alternaria tenuissima* (JB217).

The nematocidal effect of the fungal filtrates differed significantly between the nematode species (χ^2^(3) = 8.30, p = 0.040; Fig. 3). There was also a significant difference in efficacy between the filtrate treatment means (χ^2^(4) = 109.93, p < 0.001; Fig. 3). Filtrate from *F. acaciae-mearnsii* JB201 immobilized more *G. ellingtonae* J2 compared to the other fungi and the water control (χ^2^(4) = 31.142, p < .001; Fig. 3A). *Alternaria tenuissima* JB217 filtrate resulted in lower *G. ellingtonae* J2 mobility (~60%) than *F. acaciae-mearnsii* JB201 (~90%) but caused significantly higher immobility than *T. virens* JB98, *P. lilacinum* JB209, and the water control. Filtrates from *P. lilacinum* JB209 and *T. virens* JB98 had little impact on *G. ellingtonae* J2 mobility.

In the *M. chitwoodi* J2 assay, *F. acaciae-mearnsii* JB201 and *A. tenuissima* JB217 had similar impacts on J2 mobility, with ~ 100% of *M. chitwoodi* J2 being immobilized compared to *P. lilacinum* JB209, *T. virens* JB98, and the control (χ^2^(4) = 29.982, p < 0.001; Fig. 3B). Similarly, no *M. hapla* J2 were mobile after exposure to filtrates from *A. tenuissima* JB217 and *F. acaciae-mearnsii* JB201, while mobility rates caused by *T. virens* JB98 and *P. lilacinum* JB209 were comparable to the control (χ^2^(4) = 29.12, p < 0.001; Fig. 3C). The most active fungal filtrate against *M. incognita* J2 was *F. acaciae-maernsii* JB201 with a 100% reduction in mobility (χ^2^(4) = 26.174, p < 0.001; Fig. 3D). The other fungal filtrates resulted in *M. incognita* J2 motility rates similar to the water control.

## Discussion

This study evaluated filtrates from several fungi for their capability to suppress hatch of *H. gycines* and *G. ellingtonae* eggs and/or immobilize J2 of *G. ellingtonae* and three species of *Meloidogyne*. Initially, 42 fungal strains were screened against *H. glycines* and *G. ellingtonae* eggs and six were further evaluated against other PPN and life stages. The antagonistic fungi were selected not only based on their nematocidal activity but also based on the host from which they were isolated. This was done to limit the chances of selecting a specialist antagonist. The combined results demonstrated that fungal filtrates from *F. acaciae-mearnsii* JB201, and to a lesser extent *A. tenuissima* JB217, could potentially have broad applications in managing PPNs.

*Alternaria tenuissima* JB217 was selected because of the strong biocontrol efficacy observed in the initial screening assay. *Aureobasidium* sp. JB70 was retained because it was only recovered from *G. ellingtonae* cysts and members of the genus have been demonstrated to have biocontrol activity in other studies (Di Francesco et al., 2020). *Fusarium acaciae-mearnsii* JB201 and *F. proliferatum* JB173 were included because of their strong nematocidal activity in the preliminary screening, but also because members of *Fusarium* were among the most frequently isolated fungi from cyst nematodes (Jumbam et al., 2024). *Trichoderma virens* JB98 was chosen because strains of this species act as mycoparasites of plant pathogenic fungi like the cotton seed disease fungus (Howell, 2006). Species of *Trichoderma* that have been widely used for biocontrol include *T. asperellum, T. hamatum, T. harzianum, T. longibrachiatum, T. koningii, T. polysporum*, and *T. viride* (Anwar et al., 2023; Caracciolo et al., 2023; Di Marco et al., 2022; Yao et al., 2023). Although *P. lilacinum* JB209 did not have strong nematocidal effects on nematode eggs in the preliminary screening stage, it was included in subsequent experiments to serve as a positive control. Strains of this fungus are produced by Bayer and Certis Biologicals as bionematicides, and *P. lilacinum* strains have been shown to have biocontrol activity (Dahlin et al., 2019; Yu-huan et al., 2022).

*Alternaria tenuissima* JB217 filtrates reduced egg viability (~90%) compared to the other fungi. *Alternaria tenuissima* has been associated with foliar spot diseases in *Amygdalus triloba* (Chen et al., 2021), *Angelica dahurica* (Han et al., 2021), *Beta vulgaris* (Khan et al., 2020; Manea et al., 2023), *Paeonia lactiflora* (Sun and Huang, 2017), and *Solanum melongena* (Nasehi et al., 2012). It was recently reported to cause leaf spots on *Aloe barbadensis* in China (Ahmad et al., 2024). Our study is the first to link this fungus with biocontrol activity against PPNs. We found *A. tenuissima* JB217 filtrate to immobilize J2 in all of the PPNs evaluated, except for *M. incognita*. This filtrate also reduced *H. glycines* egg viability by ~80% in vitro. Filtrate from *T. virens* JB98 had a similar toxic effect on *H. glycines* eggs as *A. tenuissima*, but showed little toxicity against *G. ellingtonae* eggs. This suggests that this fungus could be specific in its nematocidal capability, being more active on *H. glycines* than *G. ellingtonae* eggs. This result corroborates studies involving plant growth–promoting rhizobacteria in which *Bacillus velezensis* had different levels of nematocidal activity against *M. incognita* and *H. glycines* J2 (Xiang et al., 2017a, 2017b). Fermented broth from *T. longibrachiatum* T6 had a strong lethal effect on the *H. avenae* eggs and on J2 in wheat (Zhang et al., 2020). *Trichoderma citrinoviride* Snef1910 and *P. lilacinum* AUMC 10149 have also been reported to kill *M. incognita* J2 (Fan et al., 2020; Isaac et al., 2021).

*Fusarium proliferatum* JB173 and *Aureobasidium* sp. JB70 reduced *G. ellingtonae* egg viability by 60 and 70%, respectively, in vitro and would be ideal candidates for future studies. Crump and Flynn (1995) reported biocontrol efficacy of *F. oxysporium* and *F. sambucinum* strains against *G. rostochiensis* and *G. pallida*. Microscopic observations of assayed eggs indicated that *F. proliferatum* JB173 and *P. lilacinum* JB209 degraded the nematode eggshell during the antagonism. This was not observed for other fungi that were screened, and we suspect the production of chitinolytic and proteolytic compounds. Dackman et al. (1989) found similar results in in vitro bioassays of *Pochonia chlamydosporia* and *Verticillium suchlasporium* on eggs of *H. avenae*. They concluded that these fungi break down nematode eggs through the production of chitinases and proteolytic enzymes. *Fusarium acaciae-mearnsii* JB201 filtrates immobilized significantly more *G. ellingtonae, M. chitwoodi, M. hapla*, and *M. incognita* J2, than all tested fungi and the control. A previous study reported that filtrates from *Penicillium chrysogenum* and *Trichoderma* spp. killed *M. javanica* J2 in vitro (Ali et al., 2023). In the same study, *M. javanica* J2 mortality rates reached 98 and 95% for *P. chrysogenum* and *Trichoderma* spp., respectively, after 72 hr incubation.

Filtrates from *Aureobasium* sp. JB70 and *P. lilacinum* JB209 had better biocontrol efficacy against *H. glycines* and *G. ellingtonae* eggs compared to J2, suggesting that these fungi may be producing vitelline-digesting enzymes. Vitelline is the outer proteinaceous layer of the nematode egg (Bird and McClure, 1976) that is prone to fungal attack via the production of proteolytic enzymes. Filtrates from *P. chlamydosporia* were reported to suppress PPNs by removing the vitelline membrane of the eggs via the production of subtilisin-like enzymes (Ward et al., 2012). Soil treatment with *P. chlamydosporia* also reduced population densities of *G. rostochiensis* and *G. pallida* to the same degree as fosthiazate in a commercial potato field in Europe (Tobin et al., 2008).

Overall, the filtrate from *F. acaciae-mearnsii* JB201 was the most effective at immobilizing J2 of all the PPNs, as well as reducing viability of *H. glycines* eggs. Except for *M. incognita* J2, filtrates from *A. tenuissima* JB217 also had significant nematocidal activity against eggs of *H. glycines* and J2 of *G. ellingtonae, M. chitwoodi*, and *M. hapla*. *Aureobasium* sp. JB70 and *P. lilacinum* JB209 resulted in lower egg viability than J2 mobility, while *F. proliferatum* JB173 was highly effective in damaging *G. ellingtonae* eggs. This is the first study to document the nematocidal effects of *A. tenuissima* JB217, *Aureobasidium* sp. JB70, *F. acacia-mearnsii* JB201, and *F. proliferatum* JB173.

Since control options for the current management of PPNs are limited (Mondello et al., 2018), this study demonstrated that *Aureobasium* sp. JB70, *A. tenuissima* JB217, *F. acaciae-mearnsii* JB201, *F. proliferatum* JB173, and *P. lilacinum* JB209 can be potential sources of bioactive metabolites for the possible management of PPNs. In planta studies using filtrates of the above fungi will further elucidate their role in suppressing PPNs. Upscaled production of these filtrates will be required for future field trials. Additionally, the potential bioactive metabolites produced by these fungi and suspected to damage and immobilize nematode eggs and J2, respectively, are yet to be fully characterized.
